# Five new species of subgenus *Plesiominettia* (Diptera, Lauxaniidae, *Minettia*) in southern China, with a key to known species

**DOI:** 10.3897/zookeys.520.9558

**Published:** 2015-09-16

**Authors:** Li Shi, Stephen D. Gaimari, Ding Yang

**Affiliations:** 1 College of Agronomy, Inner Mongolia Agricultural University, Hohhot, Nei Mongol 010019, China; 2 California State Collection of Arthropods, Plant Pest Diagnostics Center, California Department of Food and Agriculture, Sacramento, California 95618, USA; 3 Department of Entomology, China Agricultural University, Beijing 100094, China

**Keywords:** *Minettia*, Lauxaniidae, Oriental region, species key

## Abstract

Five species of the subgenus *Plesiominettia* Shatalkin from the southern China are described as new to science: Minettia (Plesiominettia) flavoscutellata **sp. n.**, Minettia (Plesiominettia) longaciculiformis **sp. n.**, Minettia (Plesiominettia) nigrantennata **sp. n.**, Minettia (Plesiominettia) tridentata **sp. n.** and Minettia (Plesiominettia) zhejiangica **sp. n.** One species, *Minettia longistylis* Sasakawa, is transferred to the subgenus *Plesiominettia* from *Minettia* s. str. A key to separate the known species of the subgenus is presented, along with a taxonomic list of species. The type materials of the new species are deposited in the China Agricultural University, Beijing, China (CAUC).

## Introduction

The subgenus *Plesiominettia* Shatalkin, 2000, of the genus *Minettia* Robineau-Desvoidy, 1830, was erected for the type species *Minettia helvola* (Becker, 1895). In the same paper, Shatalkin (2000) transferred the following species from the subgenus *Minettia* to the subgenus *Plesiominettia*: *Minettia crassulata* Shatalkin, 1998; *Minettia divaricata* Sasakawa, 1985; *Minettia filia* (Becker, 1895); *Minettia fuscescens* Shatalkin, 1998; *Minettia gemina* Shatalkin, 1992; *Minettia gemmata* Shatalkin, 1992; *Minettia helva* Czerny, 1932; *Minettia helvola* (Becker, 1895); *Minettia ishidai* (Sasakawa, 1985); *Minettia loewi* (Schiner, 1864); *Minettia omei* Shatalkin, 1998; *Minettia punctata* Sasakawa, 1985; *Minettia styriaca* (Strobl, 1892); *Minettia tenebrica* Shatalkin, 1992. The species *Minettia longistylis* Sasakawa, 2002, is here transferred into Plesiominettia from the subgenus Minettia. The subgenera of *Minettia*, as well as the species of *Plesiominettia*, can be separated using the key in this paper.

Based on combination of the original definition by Shatalkin 2000, and the authors’ observations, *Plesiominettia* is diagnosed as follows: arista pubescent, rarely bare or short plumose, rays of arista with longest setulae longer than 1/3 height of 1st flagellomere; wing uniformly hyaline, rarely pale brown at base (in Minettia (Plesiominettia) zhejiangica sp. n.); mesonotum with 0–1+2–3 dorsocentral setae (first postsutural dorsocentral setae close to transverse scutal suture or located medially between transverse scutal suture and scutoscutellar suture); acrostichal setulae hair-like, most species with 1–2 pairs of strong setae among them, located at middle of mesonotum or in front of prescutellar acrostichal setae; male genitalia: a pair of postgonites present, postgonites rarely absent; phallic sheath absent, phallus forming a case; female terminalia: spermathecae 1+1 or 1+2. In this subgenus, the body color varies from black (most species, e.g., Minettia (Plesiominettia) divaricata, Fig. 49) to grey pruinose (e.g., Minettia (Plesiominettia) gemmata, Fig. 50) to yellow (e.g., Minettia (Plesiominettia) punctata, Fig. 51).

There are 20 known species distributed in the Palaearctic and Oriental Regions in the subgenus *Plesiominettia*, 8 of which have been found in China.

## Materials and methods

The general terminology follows Cumming and Wood (2009) and Gaimari and Silva (2010). Line diagrams were drawn using a drawing tube attached to a Nikon SMZ 1500 stereomicroscope and to a Nikon 80i compound microscope. Photographs were taken by a Nikon DS-Fi2-U3 digital camera mounted on a Nikon SMZ 1500 stereomicroscope. Genitalia preparations were made by removing and macerating the apical portion of the abdomen in cold saturated NaOH for 6 hours, then soaking in distilled water with a few drops of glacial acetic acid. After examination, the genitalia were transferred to glycerin and stored in a microvial pinned below the specimen. Specimens examined were deposited in China Agricultural University, Beijing, China (CAUC). Type specimens (see Appendix) are from the following museums:

Bernice Pauahi Bishop Museum; Honolulu, Hawai’i, USA (BPBM), Hungarian Natural History Museum; Budapest, Hungary (HNHM), Hrvatski Narodni Zooloski Muzej, Zagreb, Croatia (HZMZ), Władysław Rydzewski Museum of Natural History; University of Wrocław, Poland (MNHW), Naturhistorisches Museum; Vienna, Austria (NHMW), Naturhistorisches Museum der Benediktiner-Abtei Admont; Admont, Austria (NMBA), Osaka Museum of Natural History; Osaka, Japan (OMNH), Department of Natural History, National Museums of Scotland; Edinburgh, United Kingdon (RMSE), Slovenské Národné Muzeum; Bratislava, Slovaki (SNMC), Zoological Museum; University of Amsterdam, Amsterdam, Netherlands (ZMAN), Museum für Naturkunde; Berlin, Germany (ZMHB), Zoological Museum; Moscow State University, Moscow, Russia (ZMUM).

## Taxonomy

### Subgenus *Plesiominettia* Shatalkin, 2000

*Plesiominettia* Shatalkin, 2000: 52. Type species: *Minettia helvola* (Becker, 1895) (original designation).

### Species descriptions

#### Minettia (Plesiominettia) flavoscutellata sp. n.

Taxon classificationAnimaliaDipteraLauxaniidae

http://zoobank.org/F14CA159-188B-433D-B92A-3AAD01D18EC2

Figs 1–5 , 31–33 , 43

##### Type material.

Holotype: ♂ (CAUC), CHINA, Hubei: Shennongjia National Natural Reserve, Pingqian, 1650 m, 25. vii. 2007, Qifei Liu. Paratypes: 5 ♂♂, 7 ♀♀ (CAUC), CHINA, Hubei: same data as holotype.

##### Etymology.

Latin, *flavor*-is from the latin flavus, meaning yellow, + *scutellata* is from the latin scutellatum, meaning shield; referring to the scutellum being mostly yellow; a feminine adjective.

Diagnosis. Face slightly concave, yellowish brown to blackish brown except black ventral margin. Antenna with scape brown and pedicel brownish yellow, 1st flagellomere entirely blackish brown on outer edge but yellow on basal 1/4 on inner edge. Arista short plumose, and rays of arista with longest setulae as long as 1/2 height of 1st flagellomere. Mesonotum with weak anteriormost dorsocentral setae clearly behind transverse scutal suture. Male genitalia: surstylus with a long falcate process in lateral view; phallus wide basally and narrow apically, with a small elliptical concavity at apex. Female spermathecae 1+1, round.

##### Description.

Male. Body length 3.3–3.5 mm, wing length 3.4–3.8 mm. Female. Body length 3.6–3.9 mm, wing length 3.9–4.3 mm.

Head black. Face slightly concave, yellowish brown to blackish brown except black ventral margin, parafacial yellow except black inner margin, with a narrow pale brown medial stripe. Frons blackish brown except yellow anterior margin; ocellar triangle black; ocellar setulae developed, longer than anterior fronto-orbital setae, anterior fronto-orbital setae reclinate, shorter than posterior fronto-orbital setae. Gena yellow, about 1/6 height of eye. Antenna with scape brown and pedicel brownish yellow, 1st flagellomere entirely blackish brown on outer edge but yellow on basal 1/4 on inner edge; 1st flagellomere nearly 1.6 times longer than high; arista black, except yellow at base, short plumose; rays of arista with longest setulae as long as 1/2 height of 1st flagellomere. A blackish brown spot present between eye and base of antenna. Proboscis and palpus black.

Thorax black with sparse brownish gray pollinosity, slightly subglossy. Mesonotum with 0+3 dorsocentral setae (weak anteriormost dorsocentral setae clearly behind transverse scutal suture), acrostichal setulae in 4 rows; a pair of long acrostichal setulae present in front of prescutellar setae, prescutellar setae slightly longer than 1st postsutural dorsocentral setae; 1 strong intra-alar seta, 1 anepisternal seta, 2 katepisternal setae. Scutellum mostly yellow, except blackish brown on basal 1/4–1/3. Legs: femora black; tibiae dark yellow on basal 1/2–2/3 and blackish brown on apical 1/3–1/2; tarsi dark yellow except tarsomeres 3–5 pale brown. Fore femur with 4 posteroventral setae and 8 posterodorsal setae, fore tibia with 1 short preapical anterodorsal seta and 1 short apicoventral seta. Mid femur with 4 anterior setae and 1 apical posterior seta, mid tibia with 1 strong preapical anterodorsal seta and 1 strong apicoventral seta. Hind tibia with 1 preapical anterodorsal seta and 1 short apicoventral seta. Wing (Fig. 43) slightly yellow, hyaline; costa with 2nd (between R_1_ and R_2+3_), 3rd (between R_2+3_ and R_4+5_) and 4th (between R_4+5_ and M_1_) sections in proportion of 5.3:2:1; *r-m* at middle of discal cell; ultimate and penultimate sections of M_1_ in proportion of 1:1.1; ultimate section of CuA_1_ about 1/4 of penultimate. Halter yellow.

**Figures 1–5.**
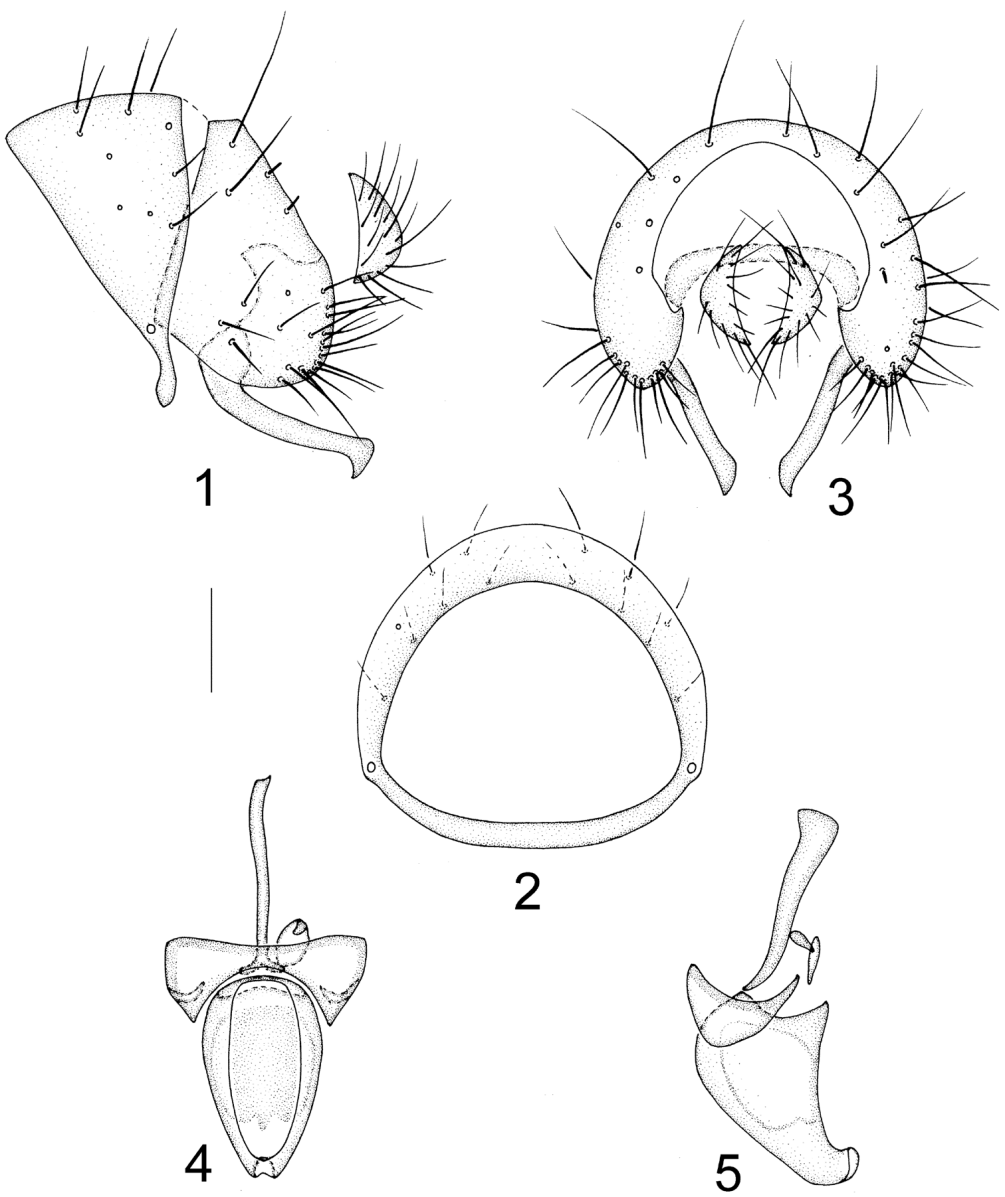
Minettia (Plesiominettia) flavoscutellata sp. n. Male. **1** syntergosternite 7+8 and epandrium, lateral view **2** syntergosternite7+8, anterior view **3** epandrial complex, posterior view **4** aedeagal complex, ventral view **5** aedeagal complex, lateral view. Scale bar: 0.1 mm.

Abdomen black with sparse brownish gray pollinosity. Male genitalia (Figs 1–5): syntergosternite 7+8 circular with dorsal setulae; epandrium broad, round apically; surstylus with a long falcate process in lateral view; hypandrium inverted–U shape, hypandrial apodeme absent; postgonite and pregonite absent; phallus wide basally and narrow apically, with a small elliptical concavity at apex; phallapodeme long, nearly as long as phallus. Female sternite 8 semicircular; spermathecae 1+1, round (Figs 31–33).

##### Remarks.

The new species is different from other species of the subgenus in the scutellum being yellow except blackish brown on basal 1/4–1/3 and the surstylus having a long falcate process in lateral view. The 1+1 female spermathecae is distinctive from the other species of the subgenus.

##### Distribution.

China (Hubei).

#### Minettia (Plesiominettia) longaciculiformis sp. n.

Taxon classificationAnimaliaDipteraLauxaniidae

http://zoobank.org/A16E8F85-E30B-43A3-A2F5-981E745BC2F4

Figs 6–10 , 34–35 , 44

##### Type material.

Holotype: ♂ (CAUC), CHINA, Zhejiang: Lin’an, Tianmushan, 19. vii. 2007, Yajun Zhu. Paratypes: 1 ♂, 6 ♀♀ (CAUC), CHINA, Zhejiang: Lin’an, Tianmushan, 18. vii. 2007, Yajun Zhu; 1 ♂ (CAUC), CHINA, Zhejiang: Lin’an, Tianmushan, Huoshandashigu, 21. vii. 2007, Yajun Zhu.

##### Etymology.

Latin, *Longi*- is from the Latin, longus, meaning long; -*acicula* is the diminutive of the Latin noun acus, meaning needle, or pin; -*formis* is from the Latin forma, meaning shape; referring to the surstylus with 2 pairs of long needle-like processes; a feminine adjective.

Diagnosis. Body yellow. Mesonotum with anteriormost dorsocentral setae situated midway between transverse scutal suture and scutoscutellar suture. Legs yellow, except brown at tip of tibiae and tarsomeres 3–5 pale brown; hind tibia with 1 weak preapical anterodorsal seta. Male genitalia: surstylus with a pair of long needle-like processes in lateral view; phallus round apically with a pair of long processes curved upward in ventral view. Female sternite 8 confluent with tergite 8, projecting on posterior margin with dense setae.

##### Description.

Male. Body length 6.5–8.0 mm, wing length 6.5–7.0 mm. Female. Body length 7.0–8.5 mm, wing length 6.6–7.0 mm.

Head yellow. Frons with ocellar triangle yellow; ocellar setulae developed, longer than posterior fronto-orbital setae, anterior fronto-orbital setae reclinate, shorter than posterior setae. Face with sparse grayish white pollinosity, without spot; gena about 1/5 height of eye. Antenna entirely yellow, 1st flagellomere 1.7 times longer than high; arista black except yellow at base, pubescent; rays of arista with longest setulae slightly shorter than 1/3 height of 1st flagellomere. Proboscis yellow except brown tip and palpus brownish yellow.

Thorax yellow with sparse grayish white pollinosity. Mesonotum with 0+3 dorsocentral setae (anteriormost dorsocentral setae situated midway between transverse scutal suture and scutoscutellar suture), acrostichal setulae in 8 rows; a pair of long acrostichal setulae present in front of prescutellar setae, prescutellar setae shorter than 1st postsutural dorsocentral setae; 1 strong intra-alar seta and 1 weak intra-alar seta situated at almost equal intervals on line between supra-alar setae and posterior dorsocentral setae; 1 anepisternal seta, 2 katepisternal setae. Scutellum yellow. Legs yellow except brown at tip of tibiae and tarsomeres 3–5 pale brown. Fore femur with 6 posteroventral setae and 9 posterodorsal setae, fore tibia with 1 short preapical anterodorsal seta and 1 short apicoventral seta. Mid femur with 8 anterior setae and 1 apical posterior seta; mid tibia with 1 strong preapical anterodorsal seta and 2 strong apicoventral setae. Hind tibia with 1 weak preapical anterodorsal seta and 1 short apicoventral seta. Wing (Fig. 44) slightly yellow, pale brown along costal margin and a brown stripe on *dm-cu*; costa with 2nd (between R_1_ and R_2+3_), 3rd (between R_2+3_ and R_4+5_) and 4th (between R_4+5_ and M_1_) sections in proportion of 5.5:1.5:1; *r-m* at middle of discal cell; ultimate and penultimate sections of M_1_ in proportion of 1:1.5; ultimate section of CuA_1_ about 1/10 of penultimate. Halter yellow.

**Figures 6–10.**
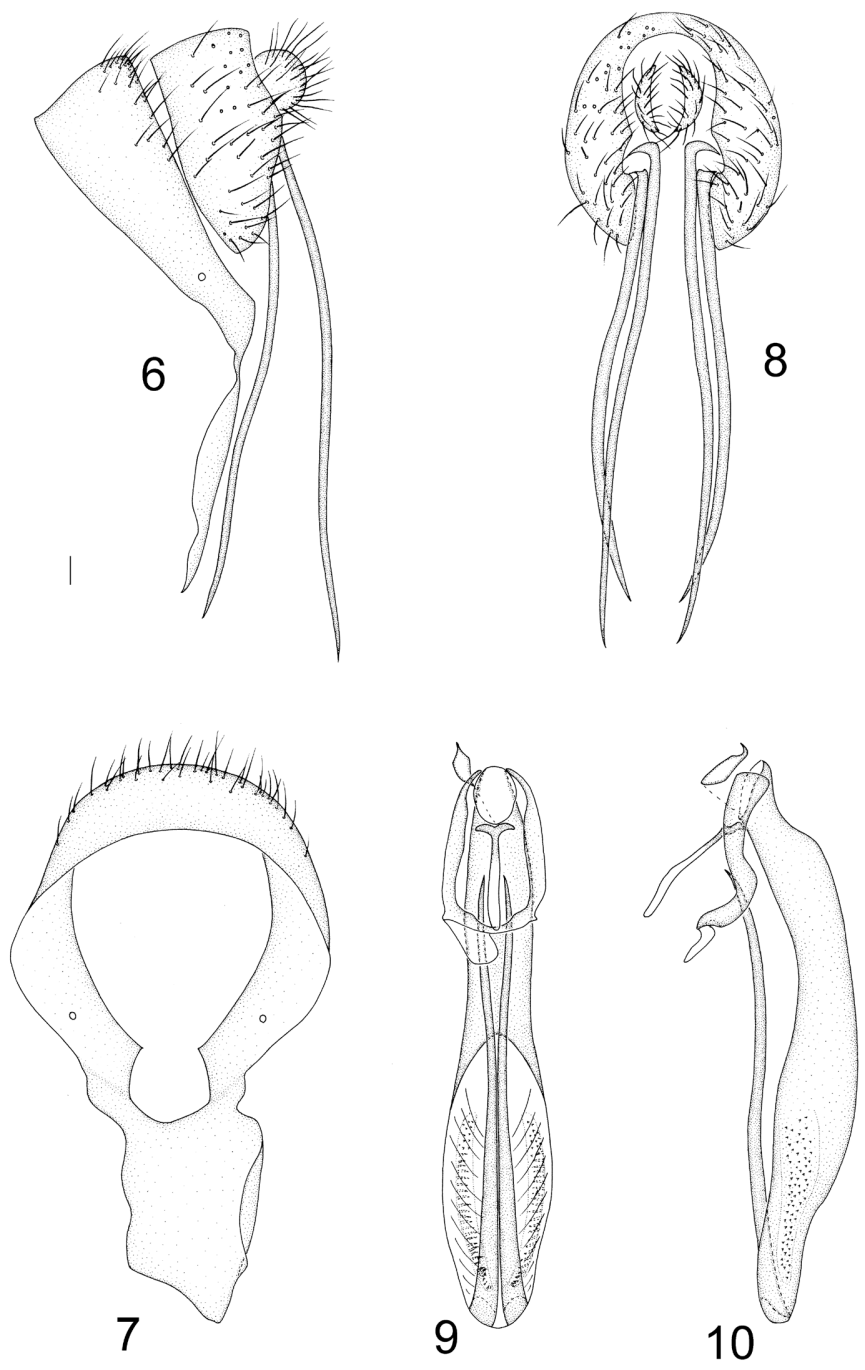
Minettia (Plesiominettia) longaciculiformis sp. n. Male. **6** syntergosternite 7+8 and epandrium, lateral view **7** syntergosternite 7+8, anterior view **8** epandrial complex, posterior view **9** aedeagal complex, ventral view **10** aedeagal complex, lateral view. Scale bar: 0.1 mm.

Abdomen yellow with sparse grayish white pollinosity. Male genitalia (Figs 6–10): syntergosternite 7+8 circular with long irregular ventral process and many dorsal setae; epandrium broad, far shorter than syntergosternite 7+8, narrow apically; surstylus with a pair of long needle-like processes in lateral view; hypandrium inverted–U shape, hypandrial apodeme indistinct; pregonite and postgonite absent; phallus slender, longer than 1/2 length of abdomen, round apically with a pair of long subuliform processes curved upward in ventral view; phallapodeme short, projecting forward. Female sternite 8 confluent with tergite 8, projecting on posterior margin with dense setae; spermathecae 1+2, nearly elliptical, each with irregular short ridges. (Figs 34–35).

##### Remarks.

The new species differs entirely from other species of the subgenus in the surstylus having a pair of very long needle-like processes and the phallus being brown, longer than 1/2 length of abdomen in ventral view.

##### Distribution.

China (Zhejiang).

#### Minettia (Plesiominettia) nigrantennata sp. n.

Taxon classificationAnimaliaDipteraLauxaniidae

http://zoobank.org/62C0D97E-160A-41B2-8800-FD17F2E614A4

Figs 11–15 , 45

##### Type material.

Holotype: ♂ (CAUC), CHINA, Hunan: Changde, Shimen, Hupingshan National Nature Reserve, Zhipeng River, 450 m, 6. vi. 2008, Li Shi.

##### Etymology.

Latin, *nigr*- is from the Latin, *nigra*, meaning black, + *antennata*, meaning antenna; referring to the blackish brown antenna; feminine adjective.

##### Diagnosis.

Arista pubescent, rays of arista with longest setulae shorter than 1/3 height of 1st flagellomere. Mesonotum with anteriormost dorsocentral setae slightly beyond suture. Legs black, except basal tip of tibia yellow, fore tarsus brown, mid and hind tarsomeres 1–2 dark yellow and tarsomeres 3–5 brown; hind femur with a row of anteroventral seta on apical half. Male genitalia: surstylus consisting of a brown knife–like apical process and a yellow bar–like apical process with setulae in lateral view; postgonite narrow basally and broad contorted apically with 2 short setulae; phallus very broad, columnar, with a pair of inner sclerites and a row of tiny spinule, visible in dorsal view.

##### Description.

Male. Body length 4.6 mm, wing length 5.0 mm.

Head blackish brown. Frons with narrow yellow margin; ocellar triangle grayish black; ocellar setulae developed, slightly longer than anterior fronto-orbital setae, anterior fronto-orbital setae reclinate, shorter than posterior fronto-orbital setae. Face and parafacial dark black; gena about 1/6 height of eye. Antenna blackish brown, 1st flagellomere 1.6 times longer than high; arista black, except pale brown at base, pubescent, rays of arista with longest setulae shorter than 1/3 height of 1st flagellomere. A blackish brown spot present between eye and base of antenna. Proboscis blackish brown and palpus black.

Thorax black with dense brownish pollinosity. Mesonotum with 0+3 dorsocentral setae (anteriormost dorsocentral setae slightly beyond suture), acrostichal setulae in 6 irregular rows, prescutellar setae as long as first postsutural dorsocentral setae; 1 strong intra-alar seta, 1 anepisternal seta, 2 katepisternal setae. Scutellum black with dense brown pollinosity. Legs black, except basal tip of tibia yellow, fore tarsus brown, mid and hind tarsomeres 1–2 dark yellow and tarsomeres 3–5 brown. Fore femur with 6 posteroventral setae and 8 posterodorsal setae, fore tibia with 1 short preapical anterodorsal seta and 1 short apicoventral seta. Mid femur with 5 anterior setae and 1 apical posterior seta; mid tibia with 1 strong preapical anterodorsal seta and 1 strong apicoventral seta. Hind femur with a row of anteroventral seta on apical half, 1 short preapical anterodorsal seta and 2 apical posterior setae, hind tibia with 1 short preapical anterodorsal seta and 1 short apicoventral seta. Wing (Fig. 45) slightly dark yellow; costa with 2nd (between R_1_ and R_2+3_), 3rd (between R_2+3_ and R_4+5_) and 4th (between R_4+5_ and M_1_) sections in proportion of 6.5:1.7:1; *r-m* beyond middle of discal cell; ultimate and penultimate sections of M_1_ in proportion of 1:1.3; ultimate section of CuA_1_ about 1/9 of penultimate. Halter yellow.

**Figures 11–15.**
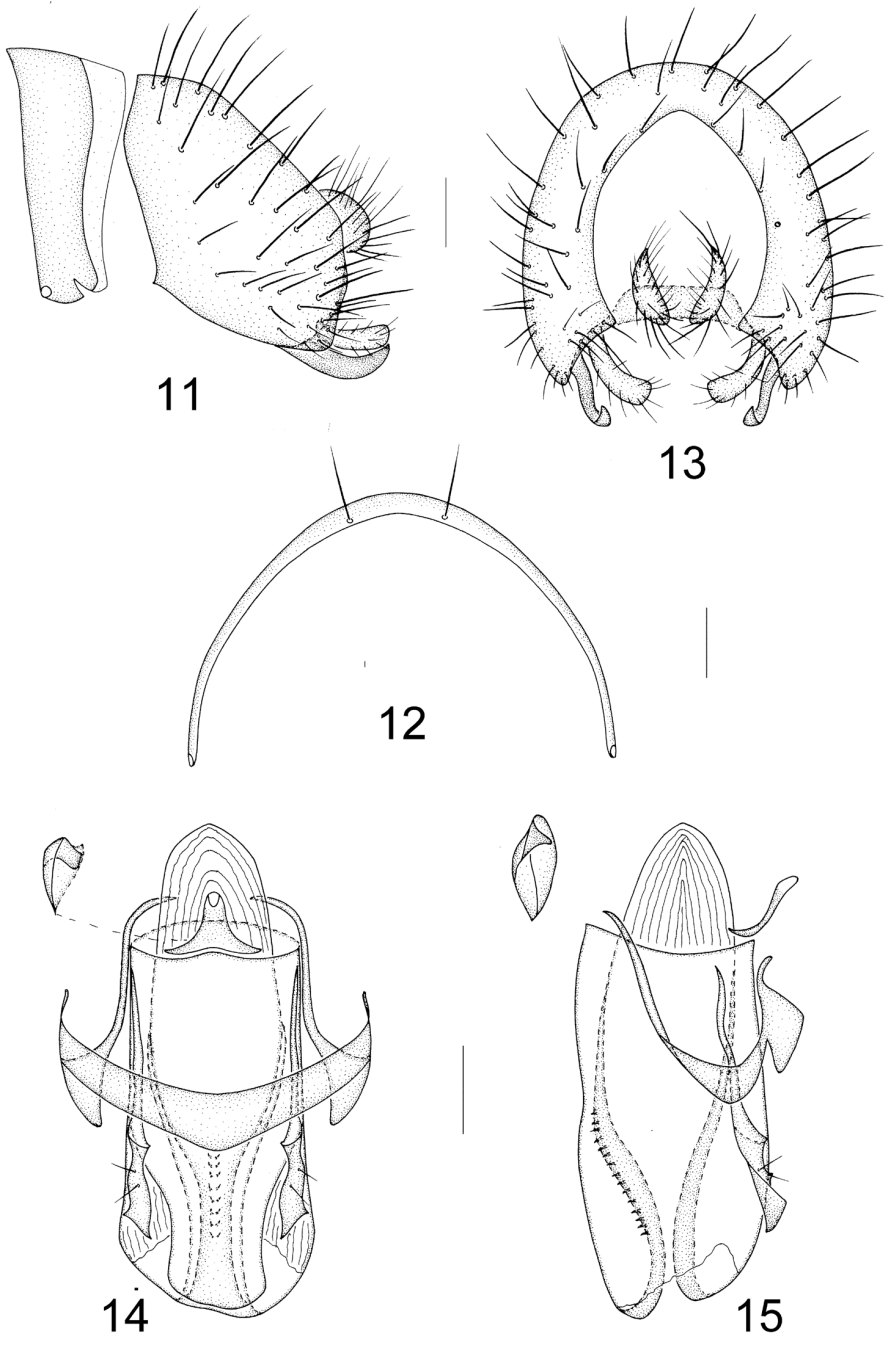
Minettia (Plesiominettia) nigrantennata sp. n. Male. **11** syntergosternite 7+8 and epandrium, lateral view **12** syntergosternite 7+8, anterior view **13** epandrial complex, posterior view **14** aedeagal complex, ventral view **15** aedeagal complex, lateral view. Scale bar: 0.1 mm.

Abdomen blackish brown with sparse brownish pollinosity, subglossy. Male genitalia (Figs 11–15): syntergosternite 7+8 semicircular with a pair of dorsal setulae; epandrium broad with long dorsal setae in lateral view; surstylus consisting of a brown knife–like apical process and a yellow bar–like apical process with setulae in lateral view; hypandrium slightly V–shaped, hypandrial apodeme very small; postgonite narrow basally, broad and contorted apically, with 2 short setulae; phallus very broad columnar, ventral sclerite narrow apically and slightly shorter than dorsal sclerite, and medial membranous section beyond base of phallus sclerites, with a pair of inner sclerites and a row of tiny spinule, visible in dorsal view; phallapodeme short, Y–shaped, slightly projecting forward.

Female. Unknown.

##### Remarks.

See Minettia (Plesiominettia) tridentata sp. n.

##### Distribution.

China (Hunan).

#### Minettia (Plesiominettia) tridentata sp. n.

Taxon classificationAnimaliaDipteraLauxaniidae

http://zoobank.org/A3170A09-8EE1-4D2A-8C39-F33835BF1F54

Figs 16–20 , 36–38 , 46

##### Type material.

Holotype: ♂ (CAUC), CHINA, Hunan: Changde, Shimen, Hupingshan National Nature Reserve, Zhipeng River, 450 m, 6. vi. 2008, Kuiyan Zhang. Paratypes: 2 ♂♂, 3 ♀♀ (CAUC), CHINA, Hunan: data same as holotype; 5 ♀♀ (CAUC), CHINA, Hunan: Changde, Shimen, Hupingshan National Nature Reserve, Zhipeng River, 450 m, 6. vi. 2008, Li Shi

##### Etymology.

Latin, *tri*-, meaning three, + *dentata*, meaning toothed; referring to a pair of subuliform inner sclerites of the phallus each with 3 acute apical teeth; a feminine adjective

##### Diagnosis.

Antennal scape blackish brown, pedicel yellow, 1st flagellomere yellow except upper and apical edges black and brown on apical half; rays of arista with longest setulae shorter than 1/4 height of 1st flagellomere. Mesonotum with anteriormost dorsocentral setae slightly beyond suture. Legs black, except mid and hind tibiae dark yellow on basal 2/3 and tarsomeres 3–5 blackish brown. Male genitalia: surstylus broad triangular in lateral view, with a tiny acute process and a small triangular apical process in ventral view. Female sternite 8 confluent with tergite 8, sternite 9 narrow triangular apically and slightly curved.

##### Description.

Male. Body length 3.8–4.3 mm, wing length 3.7–4.3 mm. Female. Body length 3.5–4.0 mm, wing length 3.9–4.4 mm.

Head blackish brown. Frons with narrow yellow margin; ocellar triangle grayish black; ocellar setulae developed, slightly shorter than anterior fronto-orbital setae, anterior fronto-orbital setae reclinate, shorter than posterior fronto-orbital setae. Face dark black and parafacial grayish black. Gena about 1/5 height of eye. Antennal scape blackish brown and pedicel yellow, 1st flagellomere yellow except upper and apical edges black and brown on apical half, 1st flagellomere 1.4 times longer than high; arista black, with microscopic setulae, and rays of arista with longest setulae shorter than 1/4 height of 1st flagellomere. A blackish brown spot present between eye and base of antenna. Proboscis blackish brown and palpus black.

Thorax black to blackish brown with dense brownish pollinosity. Mesonotum with 0+3 dorsocentral setae (anteriormost dorsocentral setae slightly beyond suture), acrostichal setulae in 8 irregular rows, prescutellar setae as long as 1st postsutural dorsocentral setae; 1 anepisternal seta, 2 katepisternal seta. Scutellum black with dense brown pollinosity. Legs black, except mid and hind tibiae dark yellow on basal 2/3 and tarsomeres 3–5 blackish brown. Fore femur with 5–6 posteroventral setae and 10 posterodorsal setae, fore tibia with 1 short preapical anterodorsal setaand 1 short apicoventral seta. Mid femur with 5–6 anterior setae, 1 apical posterior seta and 1 apicoventral seta; mid tibia with 1 strong preapical anterodorsal seta and 1 strong apical posterior seta. Hind femur with a row of anteroventral seta on apical half and 1 short preapical anterodorsal seta, hind tibia with 1 short preapical anterodorsal seta and 1 short apical posterior seta. Wing (Fig. 46) slightly dark yellow; costa with 2nd (between R_1_ and R_2+3_), 3rd (between R_2+3_ and R_4+5_) and 4th (between R_4+5_ and M_1_) sections in proportion of 5.7:1.5:1; *r-m* at middle of discal cell; ultimate and penultimate sections of M_1_ in proportion of 1:1.1; ultimate section of CuA_1_ about 1/6 of penultimate. Halter yellow.

**Figures 16–20.**
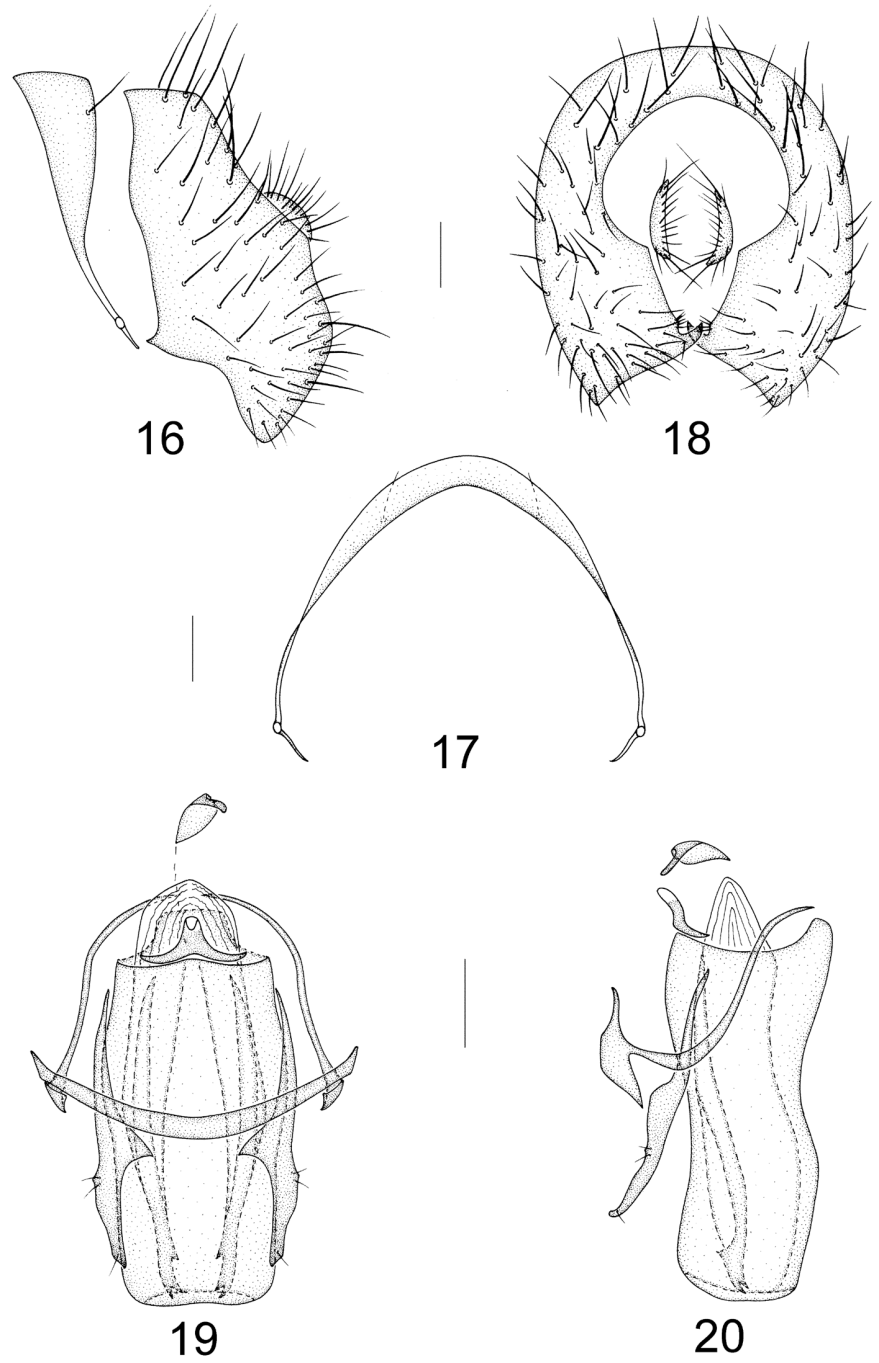
Minettia (Plesiominettia) tridentata sp. n. Male. **16** syntergosternite 7+8 and epandrium, lateral view **17** syntergosternite 7+8, anterior view **18** epandrial complex, posterior view **19** aedeagal complex, ventral view **20** aedeagal complex, lateral view. Scale bar: 0.1 mm.

Abdomen blackish brown with sparse brownish pollinosity, subglossy. Male genitalia (Figs 16–20): syntergosternite 7+8 semicircular with a pair of dorsal setulae; epandrium narrow basally with long setae and broad apically; surstylus broad triangular in lateral view, with a tiny acute process and a small triangular apical process in ventral view; hypandrium slightly V–shaped, hypandrial apodeme short; postgonite slender with a triangular medial process and two subapical setulae and one apical setula in ventral view; phallus broad columnar, with a trapezial basal process and a pair of subuliform inner sclerites each with three acute apical teeth, and medial membranous section beyond base of phallus in ventral view; phallapodeme short, Y–shaped, slightly projecting forward. Female sternite 8 confluent with tergite 8, sternite 9 narrow triangular apically and curved slightly; spermathecae 1+2, round with short brown stem (Figs 36–38).

##### Remarks.

The new species is very similar to Minettia (Plesiominettia) nigrantennata **sp. n.** from China (Hunan) in the following characters: body blackish brown; frons with yellow anterior margin and face dark black; thorax black to blackish brown with dense brownish pollinosity, mesonotum with 0+3 dorsocentral setae (anteriormost dorsocentral setae slightly beyond suture) and prescutellar setae as long as 1st postsutural dorsocentral setae; wing slightly dark yellow; abdomen blackish brown with sparse brownish pollinosity, subglossy. It can be separated from the latter by the yellow antennal 1st flagellomere, except upper and apical edges being black and brown on apical half; the legs being black, except mid and hind tibiae being dark yellow on basal 2/3. In Minettia (Plesiominettia) nigrantennata, the antennal 1st flagellomere is blackish brown and the legs are black, except basal tip of tibia is yellow, mid and hind tarsomeres 1–2 are dark yellow.

##### Distribution.

China (Hunan).

#### Minettia (Plesiominettia) zhejiangica sp. n.

Taxon classificationAnimaliaDipteraLauxaniidae

http://zoobank.org/A692F0A2-DC3E-4017-8A98-15A69A4E7C67

Figs 21–25 , 39–40 , 47

##### Type material.

Holotype: ♂ (CAUC), CHINA, Zhejiang: Longquan, Fengyangshan National Nature Reserve, Fengyang Lake, 28. vii. 2007, Yajun Zhu. Paratypes: CHINA, Zhejiang: 1 ♂, 2 ♀♀ (CAUC), Longquan, Fengyangshan National Nature Reserve, 26. vii. 2007, Yajun Zhu; 1 ♂ (CAUC), Longquan, Fengyangshan National Nature Reserve, Huangmaojian, 27. vii. 2007, Yajun Zhu; 1 ♀ (CAUC), Longquan, Fengyangshan National Nature Reserve, Huangmaojian, 29. vii. 2007, Yajun Zhu; 1 ♀ (CAUC), Longquan, Fengyangshan National Nature Reserve, Qixingtan, 1. viii. 2007, Yajun Zhu; 1 ♂, 1 ♀ (CAUC), Lin’an, Tianmushan National Nature Reserve, 19. vii. 2007, Yajun Zhu.

##### Etymology.

The new species is named after the type locality, Zhejiang Province.

##### Diagnosis.

Frons with sparse whitish gray pollinosity. Face pale brown on dorsal 1/2 and black on ventral 1/2, with sparse whitish gray pollinosity; parafacial yellow, inner margin blackish brown on ventral 1/2. Arista short plumose, rays of arista with longest setulae as long as 1/2 height of 1st flagellomere. Mesonotum with anteriormost dorsocentral setae situated on basal 1/3 between transverse scutal suture and scutoscutellar suture. Male genitalia: surstylus with a curved needle-like inner process and a geniculate outer process, acute apically.

##### Description.

Male. Body length 6.0–6.2 mm, wing length 5.9–6.3 mm. Female. Body length 5.6–6.2 mm, wing length 5.6–6.7mm.

Head pale brown. Frons with sparse whitish gray pollinosity, pale yellow on narrow anterior margin; ocellar triangle grayish black; ocellar setulae developed, longer than posterior fronto-orbital setae, anterior fronto-orbital setae reclinate, slightly shorter than posterior fronto-orbital setae. Face pale brown on dorsal 1/2 and black on ventral 1/2, with sparse whitish gray pollinosity; parafacial yellow, blackish brown on ventral 1/3, inner margin glossy black. Gena about 1/5 height of eye. Antenna brownish yellow, 1st flagellomere pale brown on apical 2/3, 1st flagellomere 2.0 times longer than high; arista black except yellow at base, short plumose, rays of arista with longest setulae as long as 1/2 height of 1st flagellomere. A grayish black triangular spot present between eye and base of antenna. Proboscis brown and palpus black.

Thorax brown with grayish pollinosity, anterior half sparse and posterior half dense. Mesonotum with 0+3 dorsocentral setae (anteriormost dorsocentral setae situated on basal 1/3 of mesonotum), acrostichal setulae in 8 rows; a pair of long acrostichal setulae present in front of prescutellar setae, prescutellar setae longer than 1st postsutural dorsocentral setae; 1 strong intra-alar seta. Anepisternum black and katepisternum brown, both with sparse grayish pollinosity; 1prescutellar setae, 2 katepisternal setae. Scutellum blackish brown with grayish pollinosity. Legs with femora black; tibiae brown except yellow on basal tip; tarsi dark yellow except tarsomeres 3–5 pale brown. Fore femur with 5–6 posteroventral setae and 10 posterodorsal setae; fore tibia with 1 short preapical anterodorsal seta and 1 short apicoventral seta. Mid femur with 6 anterior setae and 1 apical posterior seta; mid tibia with 1 strong preapical anterodorsal seta and 2 strong apicoventral seta. Hind femur with a weak preapical anterodorsal seta; hind tibia with 1 preapical anterodorsal seta and 1 short apicoventral seta. Wing (Fig. 47) slightly yellow, pale brown at base; costa with 2nd (between R_1_ and R_2+3_), 3rd (between R_2+3_ and R_4+5_) and 4th (between R_4+5_ and M_1_) sections in proportion of 5:1.5:1; *r-m* at middle of discal cell; ultimate and penultimate sections of M_1_ in proportion of 1:1.2; ultimate section of CuA_1_ about 1/6 of penultimate. Halter yellow.

**Figures 21–25.**
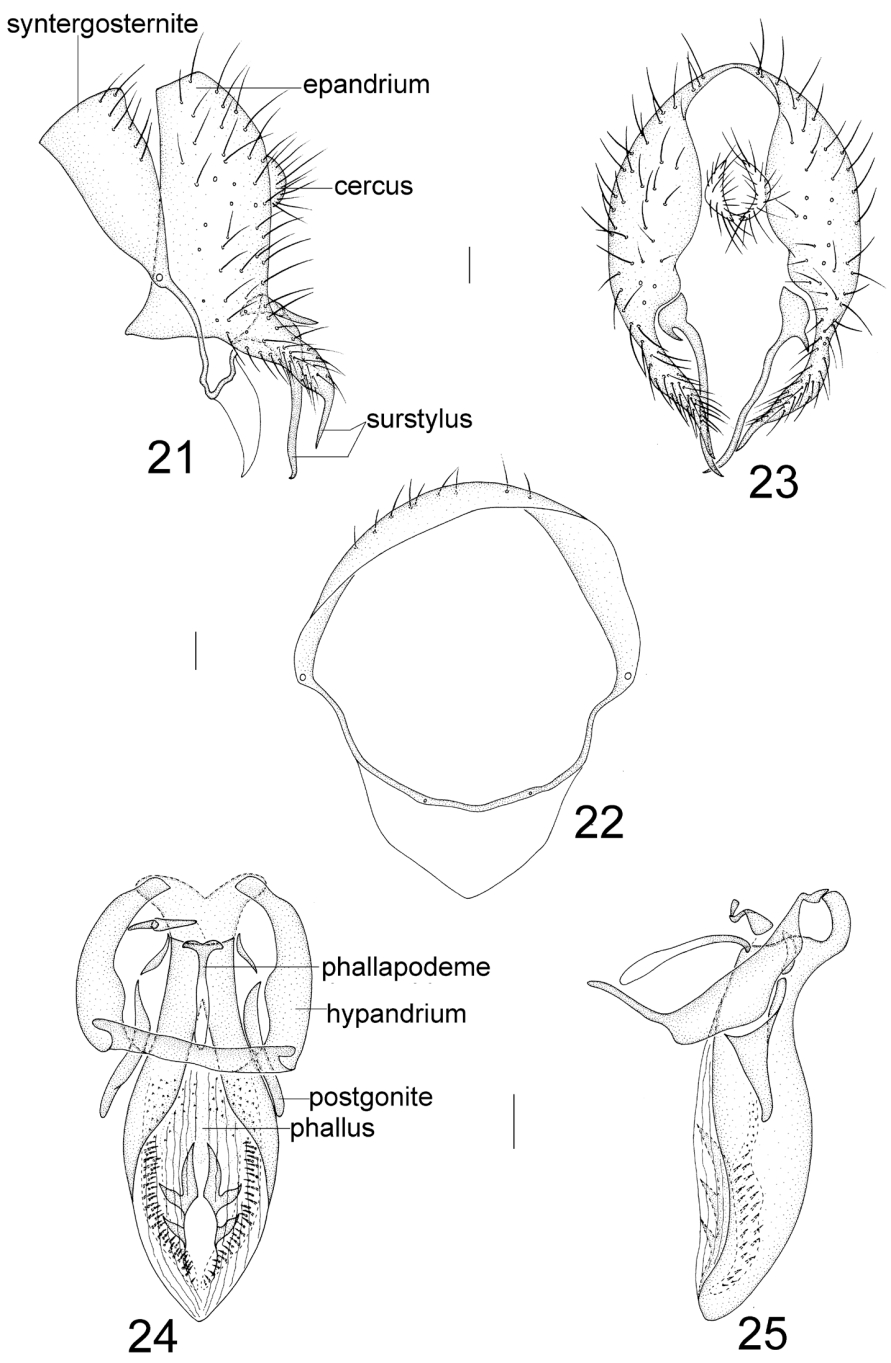
Minettia (Plesiominettia) zhejiangica sp. n. Male. **21** syntergosternite 7+8 and epandrium, lateral view **22** syntergosternite 7+8, anterior view **23** epandrial complex, posterior view **24** aedeagal complex, ventral view **25** aedeagal complex, lateral view. Scale bar: 0.1 mm.

Abdomen black with grayish white pollinosity. Male genitalia (Figs 21–25): syntergosternite 7+8 circular with a long irregular ventral process and many dorsal setulae; epandrium slender, slightly projecting at anterior ventral corner; surstylus with a curved aciculiform inner process and a geniculate outer process, acute apically; hypandrium inverted–U shape, hypandrial apodeme very short; postgonite short claviform in ventral view; phallus broad at middle, three acute processes and many spinules on membranous section in ventral view and slender and round apically in lateral view; phallapodeme small, projecting forward. Female sternite 7 rectangular and sternite 8 semicircular; spermathecae 1+2, nearly rounded and stem brown at base (Figs 39–40).

##### Remarks.

The new species is similar to Minettia (Plesiominettia) longistylis Sasakawa from China (Taiwan) in size (large), mesonotum brown with grayish pollinosity, a pair of long acrostichal setulae present in front of prescutellar setae. It can be separated from the latter by the arista being short plumose, the rays of arista having longest setulae as long as 1/2 height of antennal 1st flagellomere; the mesonotum having acrostichal setulae in 8 rows; the mid tibia having 2 posteroventral setae; the surstylus having a curved aciculiform inner process and a geniculate outer process, acute apically. In Minettia (Plesiominettia) longistylis, the arista is pubescent and the rays of arista have longest setulae as long as 1/4 height of antennal 1st flagellomere; the mesonotum has acrostichal setulae in 10 irregular rows; the mid tibia has 1 posteroventral seta; the surstylus is very long (Sasakawa 2002).

##### Distribution.

China (Zhejiang).

### Key to the subgenera of *Minettia* and the species of the subgenus *Plesiominettia*

[Modified from Stuckenberg 1971, Shatalkin 2000 and Shi and Yang 2014]

**Table:** 

1	Frons shiny and face flat; arista pubescent; mesonotum with 0–1+2–3 dorsocentral setae and 0–1+2–4 long acrostichal setulae; katepisternum with 1 strong katepisternal seta; male genitalia: phallic sheath present	subgenus ***Minettiella* Malloch**
–	Frons often dull and face slightly concave; arista pubescent or plumose; mesonotum with 0–1+3 dorsocentral setae and 0+2–3 long acrostichal setulae; katepisternum with 1 strong and 1 weak katepisternal setae; male genitalia: phallic sheath absent	**2**
2	Lower part of face with a distinct, slight and weakly round swelling on each side	**3**
–	Lower part of face without round swelling on each side	**4**
3	Basal part of wing black; arista long plumose, rays of arista with longest setulae longer than height of 1st flagellomere (rarely shorter than height of 1st flagellomere); male genitalia: two pairs of ventral hypandrial appendages (including some Palaearctic and Oriental species)	subgenus ***Frendelia* Collin**
–	Basal part of wing yellow; arista short plumose, rays of arista with longest setulae as long as 1/2 height of 1st flagellomere; male genitalia: one pair of ventral hypandrial appendages (such as *Minettia eoa* Shatalkin, 1992)	part of subgenus ***Scotominettia* Shatalkin**
4	Male genitalia: one pair of ventral hypandrial appendages (such as *Minettia austriaca* Hennig, 1951)	part of subgenus ***Scotominettia* Shatalkin**
–	Male genitalia: hypandrial appendages often transverse, bar–like, U–shaped or other shapes	**5**
5	Arista with gentle setulae, rays of arista with longest setulae as long as or shorter than1/4 height of 1st flagellomere, sometimes bare; male genitalia: phallic sheath absent (Fig. 24)	subgenus ***Plesiominettia* Shatalkin**... **6**
–	Arista pubescent or plumose, rays of arista with longest setulae longer than 1/3 height of 1st flagellomere; male genitalia: phallic sheath present (Fig. 29), square, rectangular, triangular or trapezial in shape (for example: Minettia (Minettia) lupulina (Fabricius, 1787), Figs 26–30, 41–42, 48)	subgenus ***Minettia* Robineau-Desvoidy**
6	Mesonotum with presutural dorsocentral setae	**7**
–	Mesonotum without presutural dorsocentral setae	**8**
7	Frons with dark gray stripes through *or* rows; mesonotum and scutellum dark gray; wing with dark costal margin	**Minettia (Plesiominettia) styriaca (Strobl)**
–	Frons without stripes; mesonotum yellowish brown and scutellum yellow; wing faintly yellow	**Minettia (Plesiominettia) ishidai (Sasakawa)**
8	Mesonotum without strong acrostichal setae before prescutellar setae	**9**
–	Mesonotum with 1–2 pairs of strong acrostichal setae before prescutellar setae	**13**
9	Body entirely yellow	**Minettia (Plesiominettia) filia (Becker)**
–	Body partly black or entirely brown to black (entire thorax with grey pruinose in M. kimi which is a junior synonym of Minettia (Plesiominettia) ***gemmata*** Shatalkin, fig. 50)	**10**
10	Acrostichal setulae in 8 irregular rows; male genitalia: epandrium and surstylus fused; phallus broad and truncate apically (Figs 16, 19)	**Minettia (Plesiominettia) tridentata sp. n.**
–	Acrostichal setulae in 6 rows; male genitalia: epandrium and surstylus articulate, blunt apically lateral view; phallus narrow or rounded apically	**11**
11	Face yellow with a large black round median spot above ventral margin; arista with microscopic rays; abdominal tergites 2–5 each with brownish yellow posterior margin	**Minettia (Plesiominettia) gemmata Shatalkin**
–	Face brown to black without black medial spot; rays of arista with longest setulae slightly shorter than 1/3 height of 1st flagellomere or longer than half height of 1st flagellomere; abdominal tergites 2–5 without brownish yellow posterior margin	**12**
12	Arista pubescent, rays of arista with longest setulae slightly shorter than 1/3 height of 1st flagellomere; abdomen blackish brown with sparse brownish pollinosity, subglossy; male genitalia: surstylus with narrow double processes in lateral view (Fig. 11)	**Minettia (Plesiominettia) nigrantennata sp. n.**
–	Arista plumose, rays of arista with longest setulae as long as 3/5–4/5 height of 1st flagellomere; abdominal tergites brownish yellow along medial line and dark brown to black on lateral margins; male genitalia: surstylus short and broad in lateral view (Shatalkin 2000: Fig. 95)	**Minettia (Plesiominettia) gemina Shatalkin**
13	Mesonotum with two pairs of strong acrostichal setae	**14**
–	Mesonotum with one pair of strong acrostichal setae	**16**
14	Body black except frons, face, mesonotum, scutellum and metanotum yellow; male genitalia: surstylus blunt and with hairy outgrowths (Remm and Elberg 1979: Fig. 13)	**Minettia (Plesiominettia) loewi (Schiner)**
–	Body entirely brown to black including mesonotum, scutellum and metanotum brown to black; male genitalia: surstylus short broad or in other shape	**15**
15	Arista with microscopic rays, rays of arista with longest setulae as long as 1/7 height of 1st flagellomere; frons and legs entirely brown; male genitalia: surstylus widened apically and narrow subapically, with a small concavity at middle of apical edge (Shatalkin 2000: fig. 94, Remm and Elberg 1979: Fig. 12)	**Minettia (Plesiominettia) helvola (Becker)**
–	Arista short plumose, rays of arista with longest setulae at least longer than 1/3 height of 1st flagellomere; frons yellow on ventral 1/5; legs dark brown except base of tibiae and tarsi yellowish; male genitalia: surstylus consisting of outer process narrow basally and bifurcated apically, and inner process clubbed and slender in lateral view (Sasakawa 1985: Fig. 2A–C)	**Minettia (Plesiominettia) divaricata Sasakawa**
16	Body entirely yellow (Fig. 51)	**17**
–	Body mostly brown to black	**18**
17	Mesonotum with anteriormost dorsocentral setae slightly longer than length of acrostichal setulae rows, and distinctly shorter than other dorsocentral setae; scutellum without large black lateral spots on lateral margin	**Minettia (Plesiominettia) helva Czerny**
–	Mesonotum with anteriormost dorsocentral setae distinctly stronger than other dorsocentral setae; scutellum with a pair of large black lateral spots on lateral margin (Fig. 51)	**Minettia (Plesiominettia) punctata Sasakawa**
18	Mesonotum with acrostichal setulae in 4–6 rows	**19**
–	Mesonotum with acrostichal setulae in 8–10 rows	**22**
19	Mesonotum with acrostichal setulae in 6 rows; body length 6.5 mm; male genitalia: surstylus with a pair of slender spatulate processes; phallus with 4 sharp apical processes (Shatalkin 2000: Fig. 90)	**Minettia (Plesiominettia) crassulata Shatalkin**
–	Mesonotum with acrostichal setulae in 4 rows; body length 3.3–5.0 mm; male genitalia: surstylus without a pair of slender spatulate processes; phallus without sharp apical processes	**20**
20	Arista short plumose, rays of arista with longest setulae as long as 1/2 height of 1st flagellomere; male genitalia: epandrium and surstylus articulated; surstylus with a long falcate process (Fig. 1)	**Minettia (Plesiominettia) flavoscutellata sp. n.**
–	Rays of arista with longest setulae as long as or slightly longer than basal height of arista; male genitalia: epandrium and surstylus fused; surstylus in another shape	**21**
21	Halter dark brown except stem yellowish at base; male genitalia: surstylus slender and curved, claviform, acute apically (Shatalkin 2000: Fig. 97)	**Minettia (Plesiominettia) fuscescens Shatalkin**
–	Halter entirely yellow; male genitalia: surstylus short and rounded apically (Shatalkin 2000: Fig. 96)	**Minettia (Plesiominettia) tenebrica Shatalkin**
22	Body yellow to dark yellow; male genitalia: surstylus with a pair of very long aciculiform processes (Fig. 6)	**Minettia (Plesiominettia) longaciculiformis sp. n.**
–	Body brown to black; male genitalia with short claviform process or process shaped otherwise	**23**
23	Mid tibia with 2 strong apicoventral setae; halter dark brown except stem yellowish; male genitalia: surstylus claviform narrowing gradually, with a tiny median process and tiny setulae in lateral view (Shatalkin 2000: Fig. 91)	**Minettia (Plesiominettia) omei Shatalkin**
–	Mid tibia with 1 strong apicoventral seta; halter yellow; male genitalia: surstylus in another shape, if claviform, then without a median process in lateral view	**24**
24	Arista pubescent, rays of arista with longest setulae as long as 1/4 length of 1st flagellomere; acr in 10 irregular rows; mid tibia with 1 apicoventral seta; male genitalia: surstylus with a long clubbed process, curved ventrally at tip (Sasakawa 2002: Fig. 6)	**Minettia (Plesiominettia) longistylis Sasakawa**
–	Arista short plumose, rays of arista with longest setulae as long as 1/2 length of 1st flagellomere; acrostichal setulae in 8 rows; mid tibia with 2 apicoventral setae; male genitalia: surstylus with an acute geniculate outer process and a curved needle-like inner process (Fig. 21)	**Minettia (Plesiominettia) zhejiangica sp. n.**

**Figures 26–30.**
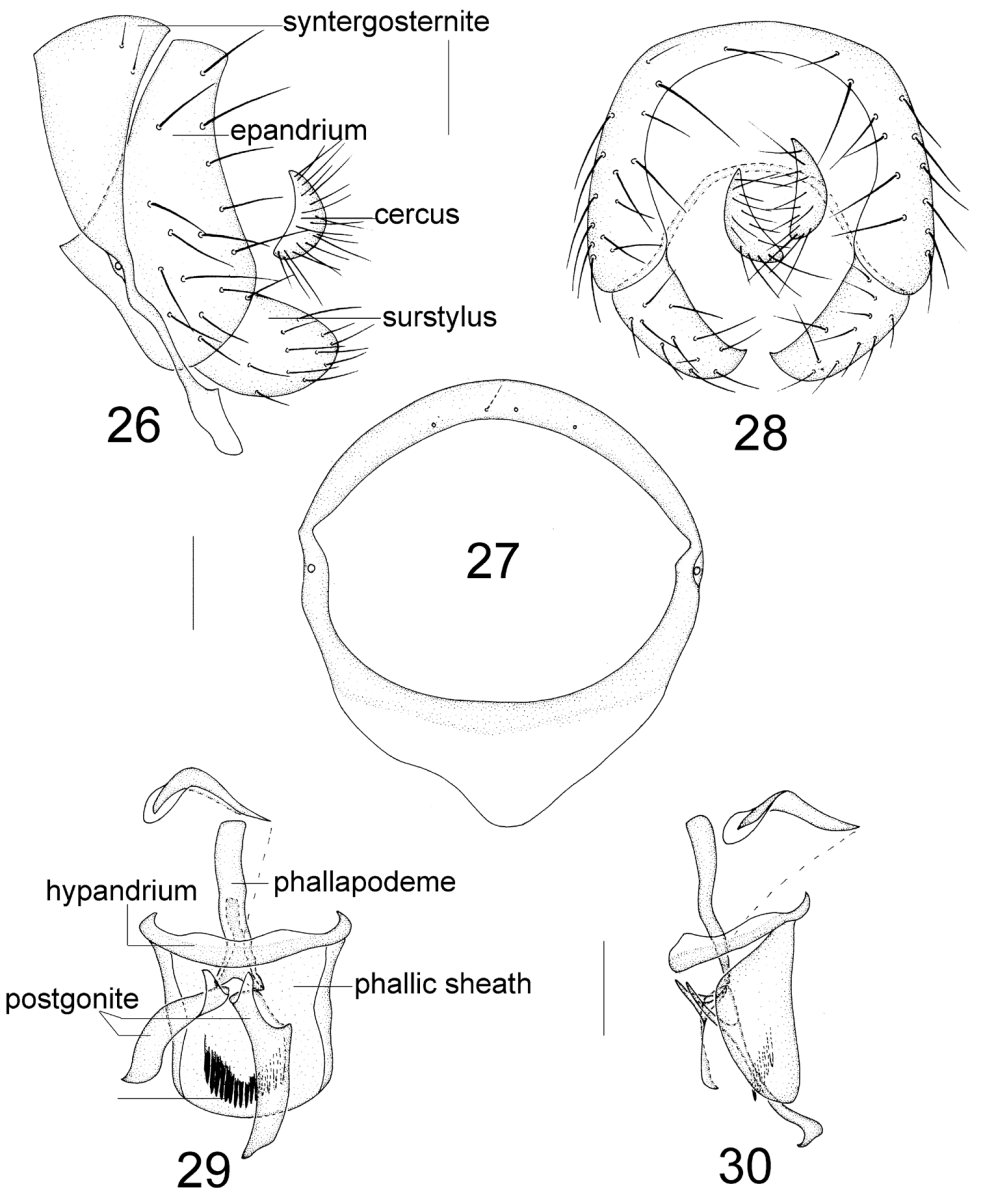
Minettia (Minettia) lupulina (Fabricius). Male. **26** syntergosternite 7+8 and epandrium, lateral view **27** syntergosternite 7+8, anterior view **28** epandrial complex, posterior view **29** aedeagal complex, ventral view **30** aedeagal complex, lateral view. Scale bar: 0.1 mm.

**Figures 31–42.**
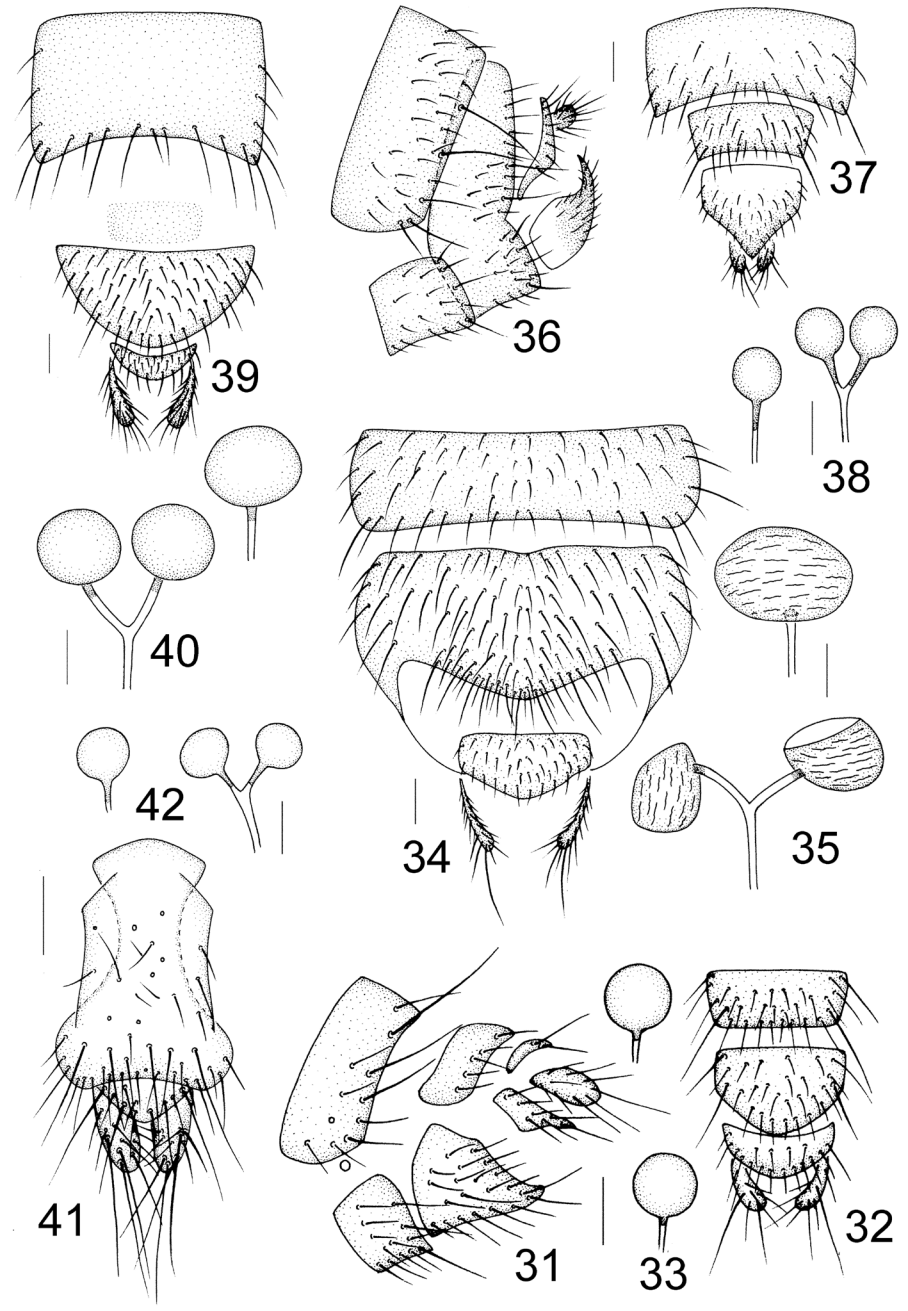
Female terminalia. Minettia (Plesiominettia) flavoscutellata sp. n. **31, 32** sternites 7–9, lateral and ventral view **33** spermathecae. Minettia (Plesiominettia) longaciculiformis sp. n. **34** sternites 7–9, ventral view **35** spermathecae. Minettia (Plesiominettia) tridentata sp. n. **36, 37** sternites 7–9, lateral and ventral view **38** spermathecae. Minettia (Plesiominettia) zhejiangica sp. n. **39** sternites 7–9, ventral view **40** spermathecae. Minettia (Minettia) lupulina (Fabricius) **41** sternites 8–9, ventral view **42** spermathecae. Scale bar: 0.1 mm.

**Figures 43–48.**
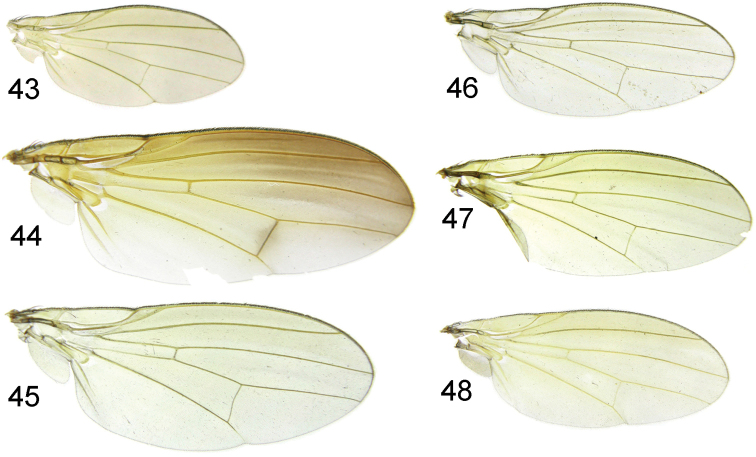
Wing. **43** Minettia (Plesiominettia) flavoscutellata sp. n. **44** Minettia (Plesiominettia) longaciculiformis sp. n. **45** Minettia (Plesiominettia) nigrantennata sp. n. **46** Minettia (Plesiominettia) tridentata sp. n. **47** Minettia (Plesiominettia) zhejiangica sp. n. **48** Minettia (Minettia) lupulina (Fabricius).

**Figures 49–51.**
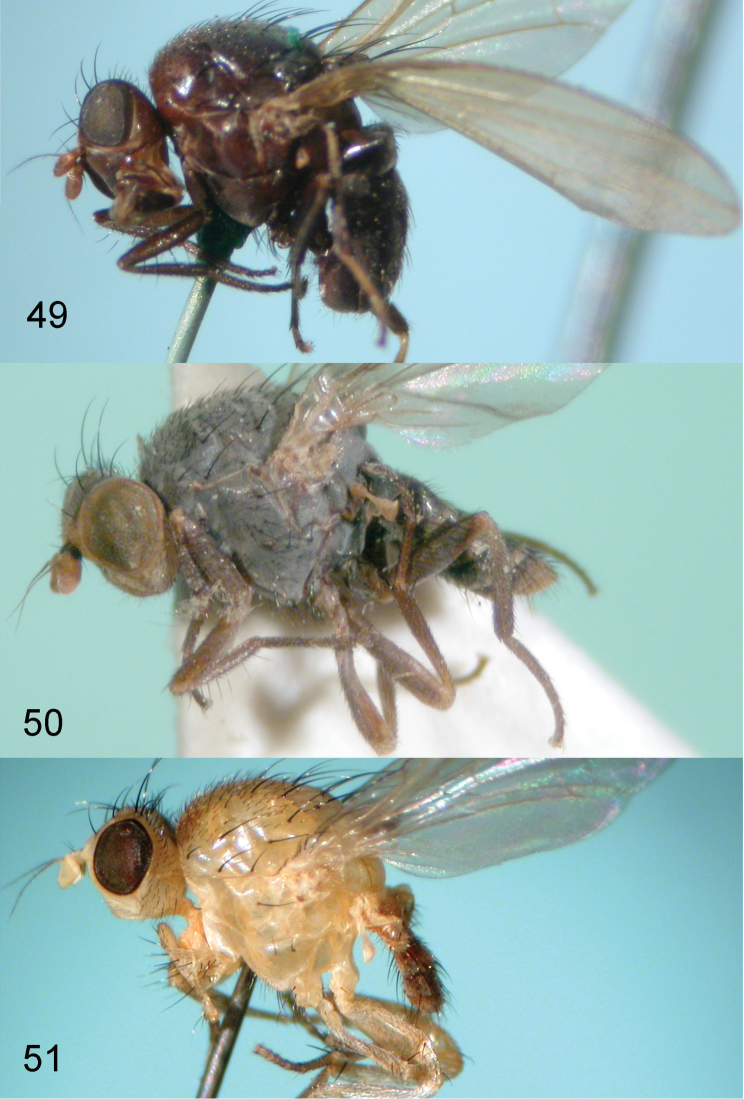
Habitus. **49** Minettia (Plesiominettia) divaricata Sasakawa, holotype male, OMNH **50** Minettia (Plesiominettia) gemmata Shatalkin (paratype female of junior synonym *Minettia kimi* Sasakawa & Kozanek, OMNH) **51** Minettia (Plesiominettia) punctata Sasakawa, holotype male, OMNH.

## Supplementary Material

XML Treatment for Minettia (Plesiominettia) flavoscutellata

XML Treatment for Minettia (Plesiominettia) longaciculiformis

XML Treatment for Minettia (Plesiominettia) nigrantennata

XML Treatment for Minettia (Plesiominettia) tridentata

XML Treatment for Minettia (Plesiominettia) zhejiangica

## Appendix

**Genus *Minettia* Robineau-Desvoidy, 1830**

**Subgenus *Plesiominettia* Shatalkin, 2000**

1. Minettia (Plesiominettia) crassulata Shatalkin, 1998a: 61. Holotype male, ZMAN. Type locality: China, Sichuan, Mt. Omei. Palaearctic: Russia. Oriental: China (Sichuan).

2. Minettia (Plesiominettia) divaricata Sasakawa, 1985: 5. Holotype male, OMNH. Type locality: Japan, Mie Prefecture, Osugidani. Palaearctic: Japan (Hokkaido, Kyushu).

3. Minettia (Plesiominettia) filia (Becker, 1895: 237) (*Sapromyza*). Syntypes female, possibly HZMZ and/or ZMHB. Type locality: Croatia, Dalmatia; Poland (“Dalmatien und Polen”). Palaearctic: Britain, Croatia, Czech Republic, Finland, Germany, Hungary, Ireland, Norway, Poland, Romania, Russia, Slovakia, Spain, Switzerland. [Note: the syntypes were in the collections of Langhoffer and Schnabl; most of the Langhoffer collection is in HZMZ, but some specimens are in ZMHB; the Schnabl collection was reported by Šifner (2008) as destroyed in WWII according to A. A. Stackelberg.]. Combination Papp, 1978: 223.

*Minettia dissimilis* Collin, 1966: 144. Holotype female, RSME. Type locality: Scotland. Dumbarton, Bonhill. Synonymy Shatalkin, 1998b: 815.

4. Minettia (Plesiominettia) flavoscutellata **sp. n.** Holotype male, CAUC. Type Locality: China, Hubei Province, Shennongjia National Natural Reserve, Pingqian. Oriental: China (Hubei).

5. Minettia (Plesiominettia) fuscescens Shatalkin, 1998b: 812. Holotype male, ZMUM. Type locality: Japan, Honshu, Nagano Prefecture, Chino-Shi. Palaearctic: Japan (Honshu, Nagano-Ken, Chino-Shi).

6. Minettia (Plesiominettia) gemina Shatalkin, 1992: 83. Holotype male, ZMUM. Type locality: Russia, Primorsky Krai, Ussuri District, Kamenushka. Palaearctic: Russia, Korea.

*Minettia tarsata* Sasakawa & Kozánek, 1995: 327. Holotype male, SNMC. Type locality: North Korea, Myohyangsan Mts., 5 km SW of Hyangsan. Synonymy Shatalkin, 1998b: 815.

7. Minettia (Plesiominettia) gemmata Shatalkin, 1992: 83. Holotype female, ZMUM. Type locality: Russia, Primorsky Krai, Ussuri District, Kamenushka. Palaearctic: Russia, Korea.

*Minettia kimi* Sasakawa and Kozánek, 1995: 323. Holotype female, SNMC. Type locality: North Korea, Ryongaksan Mts., 10 km W of Pyongyang. Synonymy Shatalkin, 1998b: 814.

8. Minettia (Plesiominettia) helva Czerny, 1932. Syntypes, 3 male and 2 female, NHMW. Type locality: “Unterlaufe des Amur” (=Russian Far East). Palaearctic: Russia.

9. Minettia (Plesiominettia) helvola (Becker, 1895: 220) (*Sapromyza*). Syntypes male and female, HNHM. Type locality: Hungary; Russia. Palaearctic: Austria, Czech Republic, Estonia, Hungary, Latvia, Liechtenstein, Russia, Slovakia, Switzerland. [Note: the syntypes were in the collections of Thalhammer and Schnabl; most of the Thalhammer collection is in HNHM; the Schnabl collection was reported by Šifner (2008) as destroyed in WWII according to A. A. Stackelberg.]. Combination Czerny, 1932: 25.

10. Minettia (Plesiominettia) ishidai (Sasakawa, 1985: 2) (*Prorhaphochaeta*). Holotype female, OMNH. Type locality: Japan, Hokkaido, Yukomanbetsu, Mt. Daisetsu. Palaearctic: Japan (Honshu), Russia.

11. Minettia (Plesiominettia) loewi (Schiner, 1864: 104) (*Sapromyza*). Replacement name for *Sapromyza bicolor* Loew, 1858. Palaearctic: Austria, Czech Republic, Finland, France, Germany, Hungary, Japan, Latvia, Lithuania, Poland, Romania, Russia, Slovakia, Switzerland. Combination Czerny, 1932: 25.

*Sapromyza bicolor* Loew, 1858: 12. Syntypes male and female, possibly MNHW and/or ZMHB (Scholtz collection). Type locality: “Silesia” (=region of Central Europe, mostly Poland, partly Czech Republic and Germany). Preoccupied by Macquart, 1835: 403.

12. Minettia (Plesiominettia) longaciculiformis **sp. n.** Holotype male, CAUC. Type locality: China, Zhejiang Province, Lin’an, Tianmushan. Oriental: China (Zhejiang).

13. Minettia (Plesiominettia) longistylis Sasakawa, 2002: 45. Holotype male, BPBM. Type locality: China, Taiwan, Mt. Alishan. Oriental: China (Taiwan). New subgenus combination.

14. Minettia (Plesiominettia) nigrantennata **sp. n.** Holotype male, CAUC. Type locality: Chian, Hunan Province, Changde, Shimen, Hupingshan National Nature Reserve, Zhipeng River. Oriental: China (Hunan).

15. Minettia (Plesiominettia) omei Shatalkin, 1998a: 61. Holotype male, ZMAN. Type locality: China, Sichuan, Mt. Omei, (between Oingyin and Chunyang). Palaearctic: Russia. Oriental: China (Sichuan).

16. Minettia (Plesiominettia) punctata Sasakawa, 1985: 5. Holotype male, OMNH. Type locality: Japan, Kyushu, Fukuoka Prefecture, Aburayama. Palaearctic: Russia, Japan (Honshu, Kyushu).

17. Minettia (Plesiominettia) styriaca (Strobl, 1892) (*Sapromyza*). Holotype female, NMBA. Type locality: Austria, Natterriegel, near Admont. Palaearctic: Austria, Finland. Combination with *Prorhaphochaeta* Czerny, 1932: 31. Combination Collin 1948: 225 (although the species was not mentioned, the genus *Prorhaphochaeta* was synonymized under *Minettia*).

18. Minettia (Plesiominettia) tenebrica Shatalkin, 1992: 84. Holotype male, ZMUM. Type locality: Russia, Primorsky Krai, Ussuri District, Kamenushka. Palaearctic: Russia.

19. Minettia (Plesiominettia) tridentata **sp. n.** Holotype male, CAUC. Type locality: China, Hunan Province, Changde, Shimen, Hupingshan National Nature Reserve, Zhipeng River. Oriental: China (Hunan).

20. Minettia (Plesiominettia) zhejiangica **sp. n.** Holotype male, CAUC. Type locality: China, Zhejiang Province, Longquan, Fengyangshan National Nature Reserve, Fengyang Lake. Oriental: China (Zhejiang).
